# Imaging transcriptomics in basal cell carcinoma: Coupling in vivo tumor morphology with gene expression

**DOI:** 10.1016/j.xjidi.2026.100519

**Published:** 2026-07-28

**Authors:** Kevin Jacobsen, Olivia Luxford Meyer, Stine Bøttcher Jacobsen, Vinzent Kevin Ortner, Silje Haukali Omland, Katrine Elisabeth Karmisholt, Martin Glud, Stine Regin Wiegell, Aditi Sahu, Cyril Maliakal, Niels Morling, Peter Alshede Philipsen, Jeppe Dyrberg Andersen, Merete Haedersdal

**Affiliations:** 1 Department of Dermatology, Copenhagen University Hospital–Bispebjerg and Frederiksberg, Copenhagen, Denmark; 2 Section of Forensic Genetics, Department of Forensic Medicine, Faculty of Health and Medical Sciences, University of Copenhagen, Copenhagen, Denmark; 3 Department of Clinical Medicine, Faculty of Health and Medical Sciences, University of Copenhagen, Copenhagen, Denmark; 4 Dermatology Service, Department of Medicine, Memorial Sloan Kettering Cancer Center, New York, New York, USA

**Keywords:** OCT, Skin cancer, Tape stripping, Whole transcriptome sequencing

## Abstract

Combining spatial and molecular information enhances understanding of skin cancer biology. In this exploratory study, we investigated such associations in basal cell carcinoma (BCC) using 2 non-invasive approaches: line-field confocal optical coherence tomography (LC-OCT) to assess tumor morphology and tape stripping for gene expression profiling. With development, this combined image-transcriptomic approach could contribute to understanding of BCC pathogenesis in vivo. Histology-confirmed BCCs (n = 53) and control skin were scanned with LC-OCT for 44 morphological features and sampled via tape stripping for mRNA analysis. Differentially expressed genes were identified and examined through pathway-enrichment to uncover mechanisms. Seven LC-OCT image-features were associated with BCC compared with control skin (*P* ≤ .004). Seventy-two differentially expressed genes were identified in BCC (false discovery rate <0.001). A subset of 27 differentially expressed genes correlated with 2 LC-OCT features—the millefeuille pattern and collagen alterations—showing a 3.9 - to 11.8-fold increase in gene expression levels. Pathway-enrichment analysis highlighted BCL2A1, linked to tumorigenesis, in association with the millefeuille pattern, and FMO2, involved in collagen synthesis, with collagen alterations. This imaging–transcriptomic approach confirms the feasibility of tape stripping for detecting BCC-specific gene expression and introduces LC-OCT-based imaging transcriptomics, for investigating BCC pathobiology in vivo.

## Introduction

Basal cell carcinoma (BCC) is the most common skin cancer among fair-skinned individuals (Rubin et al, 2005; Schmults et al, 2023), with an incidence that has tripled over the past 4 decades (Rogers et al, 2015; Venables and Wakkee, 2024). In the United States alone, over 2 million BCCs are diagnosed annually (Schmults et al, 2023). The rising incidence and high prevalence of BCC have driven the development of noninvasive tools in dermato-oncology, such as line-field confocal optical coherence tomography (LC-OCT), to improve patient care at the bedside (Abdalla et al, 2025; Peris et al, 2019).

LC-OCT is an established technique for supporting the diagnosis of various types of skin cancer and is now increasingly being explored in other applications. Using an 800-nm light source, LC-OCT can generate images of the upper skin layers at cellular resolution in real-time (Dubois et al, 2018b; Ferrante di Ruffano et al, 2018), with capabilities for full-lesion examination (Cappilli et al, 2022), and characterization of BCC-specific image features, including the millefeuille pattern, clefting, and collagen alterations (Cinotti et al, 2023; Ruini et al, 2021; Suppa et al, 2021). While LC-OCT can provide spatial tumor morphology information, the molecular mechanisms underlying BCC-specific imaging features remain largely unknown.

Integration of imaging with transcriptomic (mRNA) analysis can improve oncological disease characterization and deepen our understanding of the underlying biological mechanisms (Aerts et al, 2014; Li and Zhou, 2022; Liu et al, 2023; Sahu et al, 2022). The minimally invasive tape stripping method has emerged as a promising method for transcriptomic analysis of the entire lesion surfaces in various dermatologic conditions, including eczema and melanoma (Heerfordt et al, 2022; Hu et al, 2022; Sølberg et al, 2022). However, evidence on the feasibility of tape stripping for assessing the BCC transcriptome remains limited (Adase et al, 2021).

Coupling LC-OCT imaging with transcriptomic profiling could advance our understanding of BCC pathobiology. In this exploratory imaging-transcriptomics study, we investigated associations between LC-OCT-derived image features and gene expression profiles obtained by tape stripping in histologically confirmed BCCs. We also performed pathway enrichment analysis of the differentially expressed genes (DEGs) to identify biological mechanisms underlying the observed imaging features.

## Results

A total of 34 eligible patients (age 61 ±12.9 years) with 53 histology-verified BCCs (1–3 per patient) were included in the study (Supplementary Table S1).

### BCC transcriptomics

**Figure 1:**
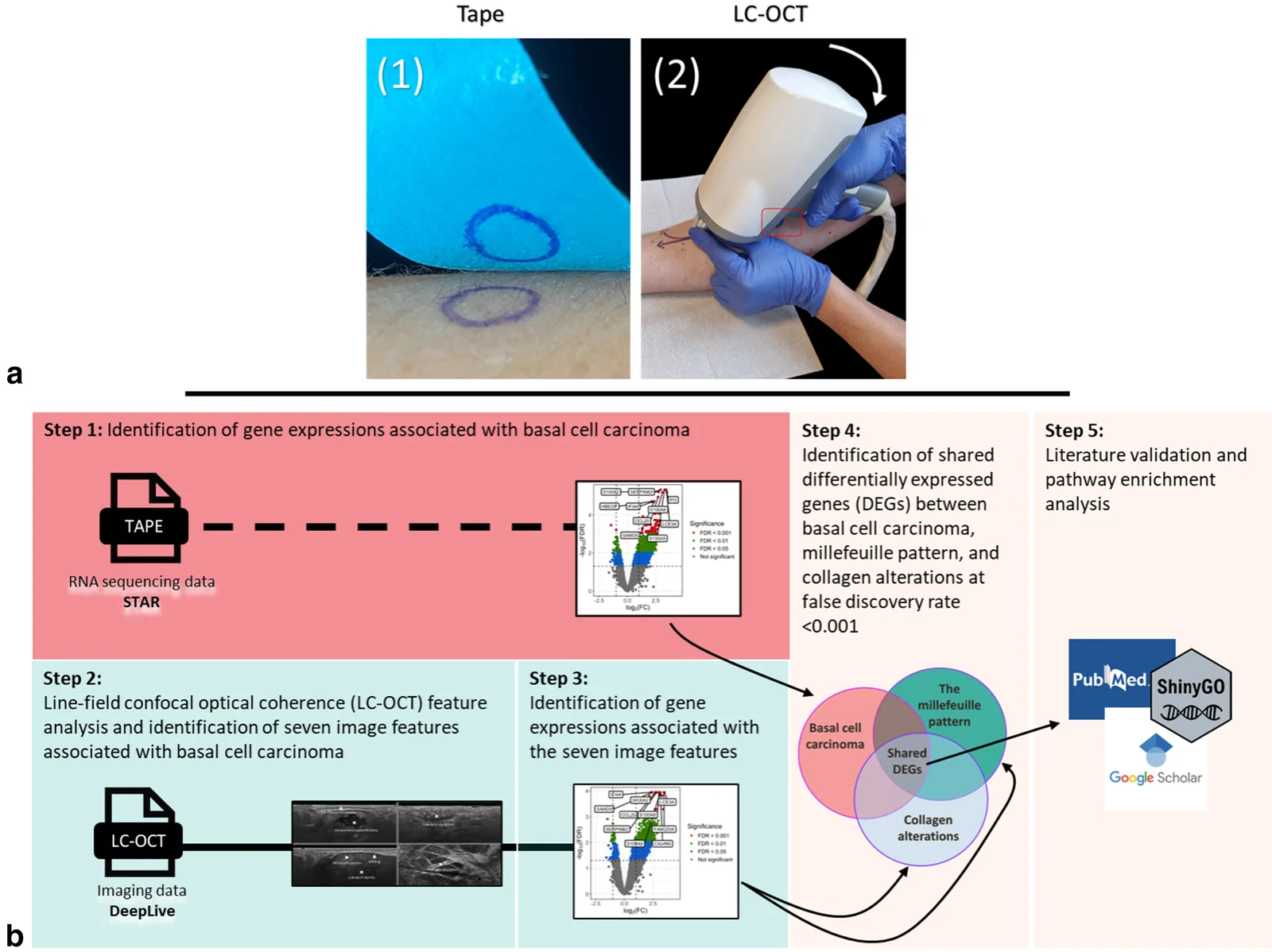
**Study design, image feature analysis, and transcriptomics analysis.** (**a**) Study design. (1) Biological material from histologically confirmed BCC and neighboring control skin was collected using tape stripping, and the mRNA was sequenced. (2) Imaging data were acquired from the same tape-stripped areas using LC-OCT. **(b)** Image feature analysis and transcriptomics analysis Step 1: DEGs associated with 53 BCC (*P* < .001) compared with 53 control skin samples were identified. Step 2: LC-OCT images of 44 skin cancer samples feature of the same BCC and control skin samples were qualitatively analyzed using the proprietary DeepLive software and statistically compared using the McNemar’s test. Seven BCC-specific image features were identified (*P* < .001). Step 3: DEGs associated with the presence of a single BCC-specific image feature FDR <0.05) compared with its absence were investigated. Step 4: Shared DEGs between BCC, millefeuille pattern, and collagen alterations at FDR <0.001 were identified. Step 5: A literature validation of the shared DEGs was conducted using PubMed and Google Scholar. GO pathways enrichment analysis of biological, cellular, and molecular pathways, as well as KEGG, was performed for shared DEGs in ShinyGo (Ge et al, 2020), an online database (https://bioinformatics.sdstate.edu/go/). BCC, basal cell carcinoma; DEG, differentially expressed genes; FDR, false discovery rate; GO, gene ontology; KEGG, kyoto encyclopedia of genes and genomes pathway; LC-OCT, line-field confocal optical coherence tomography.

Four thousand five hundred DEGs were significantly associated with BCC compared with control skin at a FDR <0.05 (step 1 in Figure 1a). A plot of these findings is presented in Supplementary Figure S1. Among these, 72 DEGs reached an FDR of <0.001; 70 had a ≥2-fold increase in expression in BCC, and 2 DEGs exhibited a decrease in expression in BCC (Supplementary Table S2). Across all 72 DEGs, expression changes ranged from a 2.8-fold decrease to an 11.2-fold increase.

### BCC-specific image features

**Figure 2:**
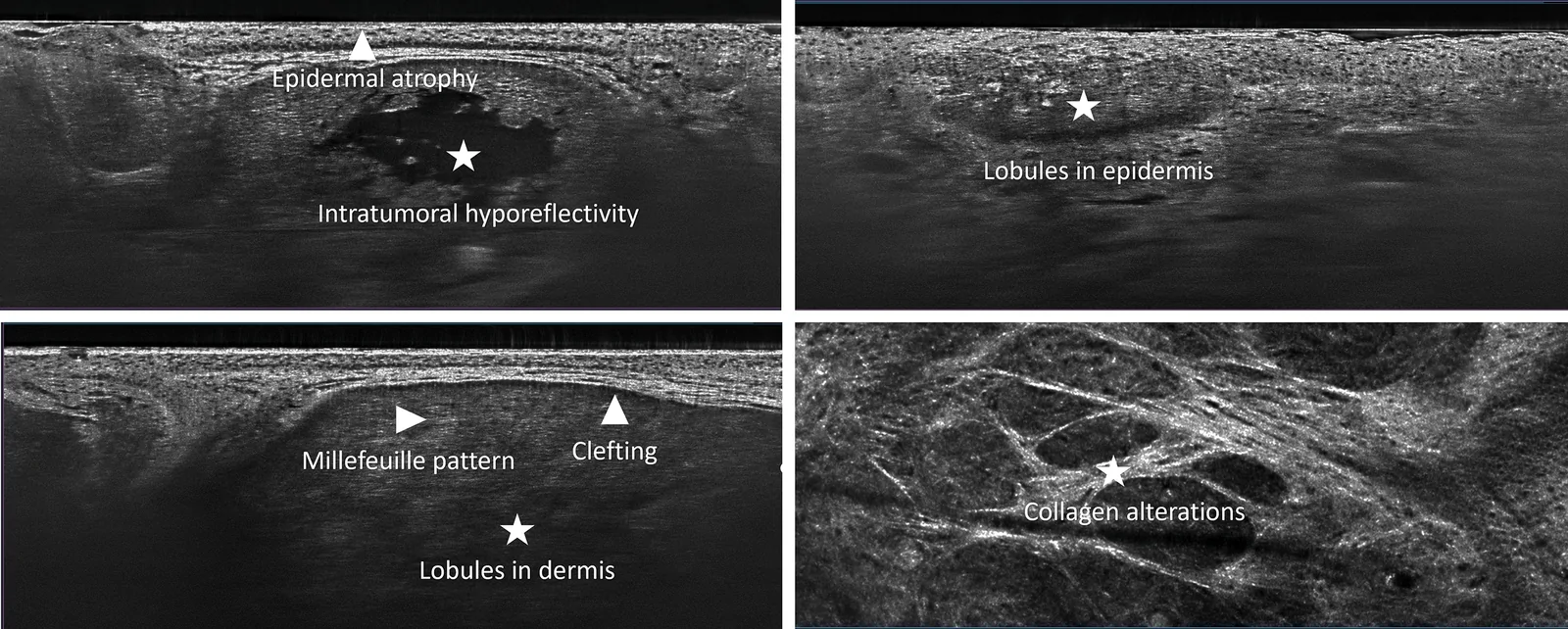
**BCC-specific image features.** Seven BCC-specific LC-OCT image features: epidermal atrophy, millefeuille pattern, lobules in the epidermis, lobules in the dermis, intratumoral hyporeflectivity, clefting, and collagen alterations. En-face images. Scale bar, 100 μm. BCC, basal cell carcinoma; LC-OCT, line-field confocal optical coherence tomography.

Seven specific LC-OCT image features (Figure 2) were significantly associated with BCC compared with control skin (Supplementary Table S3). These image features were lobules connected to the epidermis (*P* < .001), lobules not connected to the epidermis (*P* < .001), millefeuille pattern (*P* < .001), clefting (*P* < .001), intratumoral hyporeflectivity (*P* = .004), collagen alterations (*P* < .001), and epidermal atrophy (*P* < .001).

### Imaging transcriptomics DEGs

Upon investigation of DEGs in each of the 7 BCC-specific image features (step 2 and step 3 in Figure 1b), we identified: 1301 DEGs for lobules connected to the epidermis (FDR <0.05), 62 DEGs for lobules not connected to the epidermis (FDR <0.05), 3966 DEGs for the millefeuille pattern (FDR <0.05), 1814 DEGs for clefting (FDR <0.05), 1 DEG for intratumoral hyporeflectivity (FDR <0.05), 2671 DEGs for collagen alterations (FDR <0.05), and 18 DEGs for epidermal atrophy (FDR <0.05). An exemplary plot of these results can be found in Supplementary Figure S1.

At a more stringent threshold (FDR <0.001), only the millefeuille pattern and collagen alterations shared DEGs with BCC, yielding 27 shared genes (step 4 in Figure 1b; Table 1). Table 1 also includes a literature validation of the presence of the 27 shared genes in BCC.

**Table 1:** Literature validation of Differentially Expressed Genes Shared Between BCC, Millefeuille Pattern, and Collagen Alterations

Gene (full name)	Millefeuille Pattern (Fold Change [log_2_FC])	Collagen Alterations (Fold Change [log_2_FC])	Summary of Findings from Literature Search^1^
*LCE3A (Late Cornified Envelope 3A)*	11.2 × (3.48)	11.8 × (3.56)	Keratinocyte carcinoma: Potential differentiation marker (Beck et al, 2022)
*FAM220A (Family with Sequence Similarity 220 Member A)*	10.8 × (3.44)	9.3 × (3.21)	Malignant melanoma: Reported expressed (Ren et al, 2013)
*DEFB131B (Defensin Beta 131B)*	10.3 × (3.37)	12.1 × (3.61)	No reference identified
*PI3 (Peptidase Inhibitor 3/Elafin)*	9.8 × (3.28)	11.6 × (3.55)	BCC: Abnormal or disturbed squamous cell differentiation; overexpressed in eyelid BCC (Alkemade et al, 1993; MacDonald et al, 2019)
*S100A9 (S100 Calcium Binding Protein A9)*	8.4 × (3.07)	10.1 × (3.35)	Squamous cell carcinoma: Overexpressed (Shin et al, 2016). Landmark for metastasis in cutaneous tumors (Matas-Nadal et al, 2023)
*S100A8 (S100 Calcium Binding Protein A8)*	8.0 × (3.00)	10.1 × (3.34)	Squamous cell carcinoma: Overexpressed (Shin et al, 2016) Landmark for metastasis in cutaneous tumors (Matas-Nadal et al, 2023)
*SPRR2A (Small Proline Rich Protein 2A)*	N/A	8.2 × (3.05)	Actinic keratosis and squamous cell carcinoma progression: Upregulated in keratinocytes following ultraviolet radiation and in squamous cell carcinoma; consistently downregulated during actinic keratosis to squamous cell carcinoma transition, suggesting a role in tumor progression (Zou et al, 2023)
*SERPINB2 (Serpin Family B Member 2)*	7.0 × (2.80)	7.6 × (2.92)	Response to ultraviolet radiation (Majoros et al, 2019) Upregulated by GLI / HH pathway (Eichberger et al, 2006).
*SPRR2G (Small Proline Rich Protein 2G)*	N/A	7.4 × (2.89)	Implicated in skin barrier dysfunction (Zhang et al, 2019)
*CLEC2A (C-Type Lectin Domain Family 2 Member A)*	N/A	7.3 × (2.87)	Is the ligand for NK cells which function have been implicated in BCC lesions (Dachani et al, 2024; Pereira et al, 2022)
*LCE3D (Late Cornified Envelope 3D)*	N/A	7.3 × (2.86)	Late-cornified gene (Ramchatesingh et al, 2025).
*P2RY14 (Purinergic Receptor P2Y14)*	6.9 × (2.77)	N/A	Head and neck squamous cell carcinoma: Identified as a prognostic factor (Meng et al, 2021)
*CCL20 (C-C Motif Chemokine Ligand 20)*	6.6 × (2.71)	7.2 × (2.85)	Basal cell carcinoma: Overexpressed and involved in the Immune Microenvironment (Kaporis et al, 2007; Zilberg et al, 2023). Squamous cell carcinoma: Involved in tumor-associated immunosuppression (Pant et al, 2024)
*C1orf52 (Chromosome 1 Open Reading Frame 52)*	N/A	6.9 × (2.78)	No reference identified
*S100A2 (S100 Calcium Binding Protein A2)*	6.4 × (2.68)	6.8 × (2.76)	Inflammatory skin conditions: Upregulated in keratinocytes under toxic condition, psoriasis and atopic dermatitis; marker of keratinocyte damage (Yoshioka et al, 2021). Potential of cytoplasmic S100A2 overexpression as a predictor of recurrence risk (Kumar et al, 2015)
*MS4A4A (Membrane Spanning 4-Domains A4A)*	6.3 × (2.66)	N/A	Malignant melanoma: Required for Dectin-1-mediated control of metastatic spreading (Mattiola et al, 2019).
*S100A12 (S100 Calcium Binding Protein A12)*	5.9 × (2.57)	N/A	Malignant melanoma and Psoriasis: Overexpressed; interacts with RAGE enhances inflammation and lesion progression (Eckert et al, 2004)
*IL36G (Interleukin 36 Gamma)*	5.5 × (2.45)	6.6 × (2.73)	Oral squamous cell carcinoma: Pro-tumorigenic function (Chelvanambi et al, 2020)
*S100A6 (S100 Calcium Binding Protein A6)*	N/A	5.8 × (2.52)	Squamous cell carcinoma: Expressed in squamous cell carcinoma but not in basal cell carcinoma (Fullen et al, 2008; Yang et al, 2023)
*SAMD9 (Sterile Alpha Motif Domain Containing 9)*	5.3 × (2.41)	5.6 × (2.49)	No reference identified
*BCL2A1 (BCL2 Related Protein A1)*	5.2 × (2.37)	N/A	BCC: deregulated hedgehog signaling in basal cell carcinoma in mice leads to high levels of bcl-2 protein (Bigelow et al, 2004) Malignant melanoma: Amplified in approximately one-third of tumors and is necessary for growth in cell cultures (Haq et al, 2013)
*FMO2 (Flavin Containing Dimethylaniline Monoxygenase 2)*	N/A	5.0 × (2.31)	BCC: Identified among the top differentially expressed genes in BCC without PTCH1 alteration (Jasmine et al, 2024)
*WFDC12 (WAP 4-Disulfide Core Domain 12)*	N/A	4.8 × (2.25)	Atopic dermatitis: Overexpression and linked to the severity of the disease in mice (Li et al, 2023)
*IFI44 (Interferon Induced Protein 44)*	5.0 × (2.32)	4.6 × (2.21)	Head and neck squamous cell carcinoma: Overexpressed and may be involved in tumor infiltration and survival (Pan et al, 2020; Rupa et al, 2024) BCC: Upregulated (Dai et al, 2018)
*HBEGF (Heparin-Binding EGF-Like Growth Factor)*	4.5 × (2.17)	4.6 × (2.20)	Upregulated in squamous cell carcinoma and asymptomatic epidermis near BCC (Rittié et al, 2007)
*IL1RN (Interleukin 1 Receptor Antagonist)*	N/A	4.0 × (2.01)	Vulvar squamous cell carcinoma: Upregulated in patients with human papilloma virus TP53 positive tumors (Moufarrij et al, 2025). Key upstream regulator of metastasis in squamous cell carcinoma (Minaei et al, 2022)
*ADAM32 (ADAM Metallopeptidase Domain 32)*	3.9 × (1.94)	N/A	No reference identified.

Abbreviations: BCC, basal cell carcinoma; DEG, differentially expressed genes; FDR, false discovery rate; HH, hedgehog; RAGE, receptor for advanced glycation endproducts.

This table reports DEGs shared between BCC, millefeuille pattern, and collagen alterations. The genes presented have a FDR <0.001. The table is ordered by gene expression level (log_2_ fold change) between BCC and control skin. Grey shaded rows correspond to DEGs that were previously reported in BCC.

1 A literature validation of DEGs previously reported in BCC or other skin conditions was conducted using PubMed and Google Scholar. N/A indicates that DEGs had an FDR >0.001.

**Figure 3:**
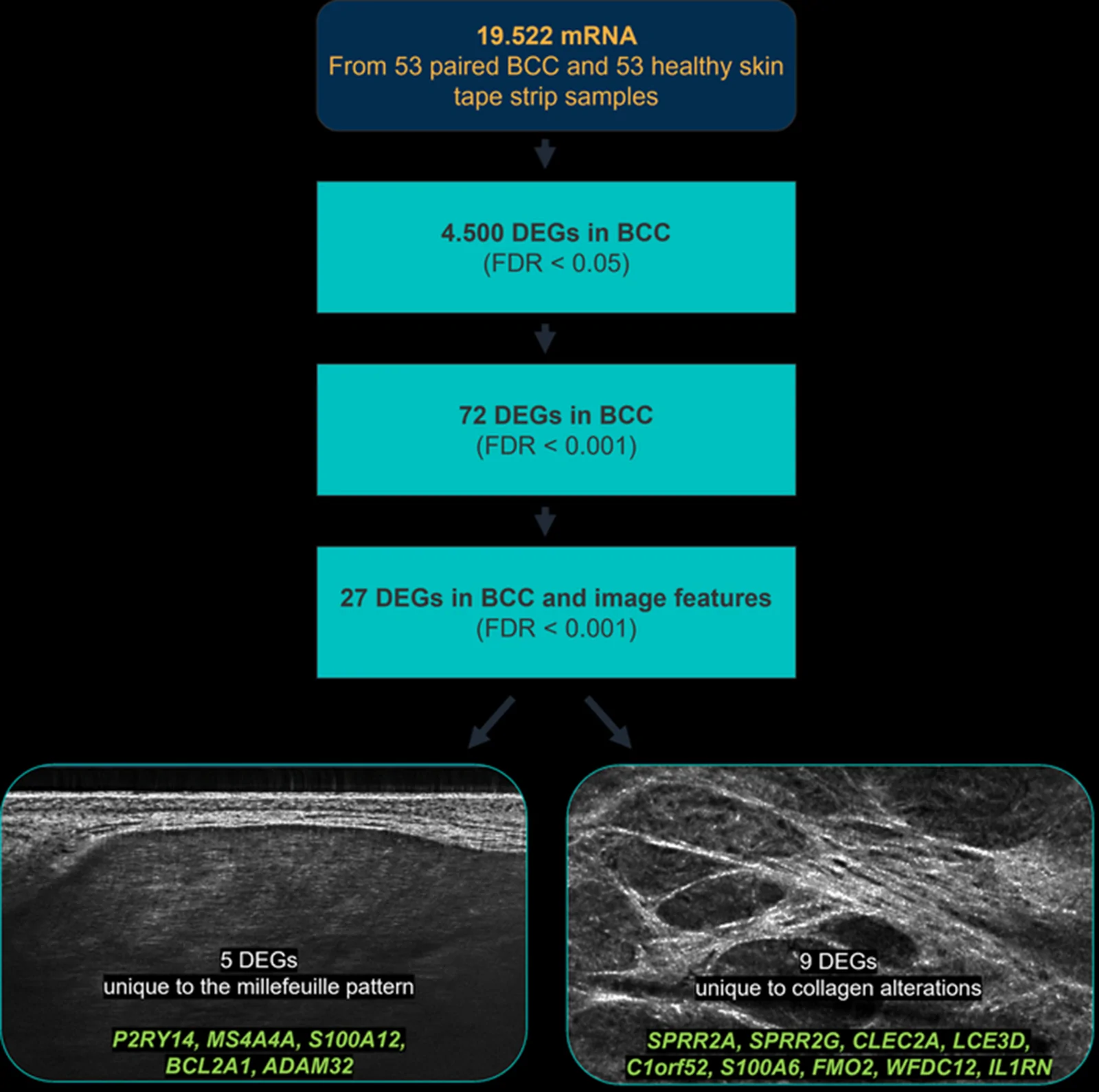
**Shared differentially expressed genes between BCC, millefeuille pattern and collagen alterations.** DEGs in BCC compared with those in control skin were identified (n = 4500, false discovery rate (FDR)/*P* < .05 and n = 72, FDR/*P* < .001). Among these, the shared DEGs between BCC and BCC-specific image features were found (n = 27 DEGs, FDR/*P* < .001). Among these, 5 DEGs were identified for the millefeuille pattern, whereas 9 DEGs were unique to collagen alterations. BCC, basal cell carcinoma; DEG, differentially expressed genes; FDR, false discovery rate.

Among these, 13 DEGs were associated with both image features, whereas 5 were specific to the millefeuille pattern (*P2RY14, MS4A4A, S100A12, BCL2A1*, and *ADAM32*), and 9 were unique to collagen alterations (*SPRR2A, SPRR2G, CLEC2A, LCE3D, C1orf52, S100A6, FMO2, WFDC12,* and *IL1RN*) (Figure 3). The expression changes of the 27 DEGs ranged from a 3.9-fold to 11.8-fold increase.

### Pathway enrichment results

Table 2 shows the enrichment results of the 27 shared DEGs between BCC, the millefeuille pattern, and collagen alterations.

**Table 2:** Pathway Enrichment Analysis of DEG for BCC-Specific Image Features

Category	Specific for Millefeuille Pattern	Specific for Collagen Alterations	Found in Both Image Features
Input DEGs (N genes)	*P2RY14, MS4A4A, BCL2A1, S100A12, ADAM32* n = 5	*SPRR2G, CLEC2A, SPRR2A, FMO2, IL1RN, LCE3D, C1orf52, S100A6, WFDC12* n = 9	*LCE3A, S100A8, S100A2, S100A9, PI3, SERPINB2, CCL20, SPRR2G, SAMD9, IL36G, CLEC2A, HBEGF, SPRR2A, FMO2, P2RY14, IL1RN, MS4A4A, LCE3D, C1orf52, DEFB131B, BCL2A1, S100A12, S100A6, ADAM32, IFI44, WFDC12, FAM220A* n = 27
KEGG pathways (N enriched pathways)	Acute myeloid leukemia (*BCL2A1*); NF-κB signaling (*BCL2A1*); Apoptosis *(BCL2A1*); Transcriptional misregulation in cancer (*BCL2A1*) n = 4	Taurine and hypotaurine metabolism (*FMO2*) n = 1	IL-17 signaling (*S100A8, S100A9, CCL20*) n = 1
GO: Molecular function (N enriched pathways)	G protein–coupled pyrimidinergic receptor activity (*P2RY14)*; RAGE receptor binding (*S100A12*); BH domain binding (*BCL2A1*); Purinergic nucleotide receptor activity (*P2RY14)* n = 5	IL-1 receptor antagonist activity (*IL1RN)*; N,N-dimethylaniline monooxygenase activity (*FMO2)*; Hypotaurine monooxygenase activity (*FMO2)* n = 3	Toll-like receptor 4 binding (*S100A8, S100A9*) - highest effect size n = 63
GO: Cellular component (N enriched pathways)	None	Cornified envelope (*SPRR2G, SPRR2A*) n = 1	Cornified envelope (*PI3, SPRR2G, SERPINB2, SPRR2A)* - highest effect size n = 3
GO: Biological process (N enriched pathways)	None	Antibacterial humoral response (*WFDC12, SPRR2A*); Keratinization (*SPRR2G, LCE3D*) n = 2	Autocrine signaling (*S100A8, S100A9)* - highest effect size n = 161

Abbreviations: BCC, basal cell carcinoma; DEG, differentially expressed genes; FDR, false discovery rate; GO, gene ontology; KEGG, kyoto encyclopedia of genes and genomes pathway; RAGE, receptor for advanced glycation endproducts.

Significant enrichment results (FDR <0.05) from the ShinyGO enrichment analysis platform (Ge et al, 2020; https://bioinformatics.sdstate.edu/go/). The input DEGs associated with each image feature or with both are shown. The DEGs that overlap with the genes in the enriched pathways are also shown. The KEGG and the GO databases (molecular function, cellular component, and biological process levels) were utilized. The top 4 pathways based on fold enrichment (indicating the magnitude of enrichment/effect size) are shown when applicable for the “millefeuille pattern” and “collagen alterations” categories, whereas a single pathway with the highest fold enrichment is shown for the “both image features” category. n represents the number of pathways identified for each assessment.

Pathway enrichment analysis revealed that the top Kyoto Encyclopedia of Genes and Genomes pathways for the millefeuille pattern DEGs included acute myeloid leukemia, NF-κB signaling, apoptosis, and transcriptional misregulation in cancer. GO molecular function terms were enriched for G protein-coupled purinergic nucleotide receptor activity, RAGE receptor binding, and BH domain binding, with no significant enrichment in the cellular component or biological process categories.

For collagen alteration DEGs, the top Kyoto Encyclopedia of Genes and Genomes pathway was taurine and hypotaurine metabolism, while GO molecular functions included IL-1 receptor antagonist and monooxygenase activities. Enriched GO cellular components and biological processes involved the cornified envelope, antibacterial humoral response, and keratinization. DEGs shared between both image features were primarily enriched in the IL-17 signaling pathway, with GO terms related to Toll-like receptor binding, cornified envelope, and autocrine signaling.

## Discussion

This study was exploratory in nature, taking advantage of coupling non-invasive optical imaging with transcriptomics to generate hypotheses; i) it suggests the feasibility of tape stripping for identifying BCC-specific gene expression, and ii) it introduces, to our knowledge, previously unreported LC-OCT-based imaging transcriptomics, potentially offering insights into BCC pathobiology in vivo. Consequently, the results suggest that 27 DEGs were shared between BCC and 2 LC-OCT image features: the millefeuille pattern and collagen alterations.

### Tape stripping for BCC transcriptomic analysis

Evidence supporting the feasibility of skin surface transcriptomics in BCC is limited, with only a single study reporting the use of “smart stickers” similar to our tape stripping method (Adase et al, 2021). In that study, several late cornified envelope (*LCE*) genes were identified among the top 12 DEGs capable of distinguishing BCC from non-cancerous skin. Consistently, our study suggested late cornified envelope genes among the top 72 DEGs in BCC compared with control skin. Combined with its known role in differentiating epithelia in response to ultraviolet damage (Jackson et al, 2005), late cornified envelope expression may serve as a reproducible, skin surface-detectable signature of BCC. In addition, Kyoto Encyclopedia of Genes and Genomes pathway analysis revealed Hedgehog (HH) pathway-related genes among the significant DEGs for BCC, including *KIF3A, EFCAB7, MGRN1, HHIP, SPOP*, and *SMURF2*. Altogether, we suggest evidence that tape stripping captures key DEGs of BCC, including late cornified envelope and DEGs related to HH pathway activity.

### Transcriptomic Insights Underlying LC-OCT Image Features in BCC

To our knowledge, this study is the first to evaluate 2 key morphologic features of BCC in relation to molecular pathway alterations, offering insights into the biological underpinnings of BCC.

The most indicative LC-OCT image feature for BCC, is the millefeuille pattern within tumor lobules (Mtimet et al, 2025). This laminated optical pattern, coined by Suppa et al, (2021), has been interpreted to correspond to the dense cellularity of BCC tumor islands observed in histopathology. While present in the majority of BCCs included (90.6%), no study has investigated whether the absence of this feature is a tumor-defining characteristic or a consequence of low signal quality.

Pathway enrichment analysis of millefeuille-specific DEGs identified pathways that may support BCC growth and survival, including acute myeloid leukemia, NF-κB signaling, apoptosis, and transcriptional misregulation in cancer. These findings suggest that *BCL2A1* may promote BCC progression through HH pathway crosstalk, apoptosis inhibition, and enhanced proliferation (Bigelow et al, 2004). Other potential therapeutic targets include *P2RY14*, implicated in

G-protein coupled receptors-signaling relevant to BCC pathogenesis (Meng et al, 2021; Zhang et al, 2024) and *S100A12*, which may contribute to pro-inflammatory RAGE-pathway-mediated mechanisms (Eckert et al, 2004).

For the collagen alteration feature, corresponding to the bright stromal rim seen in LC-OCT images (Suppa et al, 2021), DEGs were enriched in the hypotaurine-taurine metabolism pathway via *FMO2*, suggesting an antiinflammatory and tissue remodeling role (Yoshimura et al, 2023). Additional genes, such as *IL1RN* and *WFDC12* reflected opposing anti- and pro-inflammatory processes (Freeman and Buchman, 2001; Li et al, 2023), indicating that collagen alterations may influence responsiveness to immunomodulatory therapies (Sahu et al, 2022). Together, these results underscore the interplay between tumor-stroma interactions, inflammation, and potential therapeutic vulnerability in BCC.

Of the shared DEGs between BCC, millefeuille pattern, and collagen alterations (Table 2), 5 were validated in the BCC literature: *PI3* (Alkemade et al, 1993; MacDonald et al, 2019), *CCL20* (Kaporis et al, 2007; Zilberg et al, 2023), *FMO2* (Jasmine et al, 2024), *IFI44* (Dai et al, 2018), and *HBEGF* (Rittié et al, 2007). This may support the feasibility of using tape stripping for gene expression profiling in BCC.

Several DEGs, such as *SERPINB2* and *BCL2A1*, appear to be indirectly involved in BCC through dysregulation of the HH signaling pathway, a known driver of BCC (Maroufy et al, 2020).

**Figure 4:**
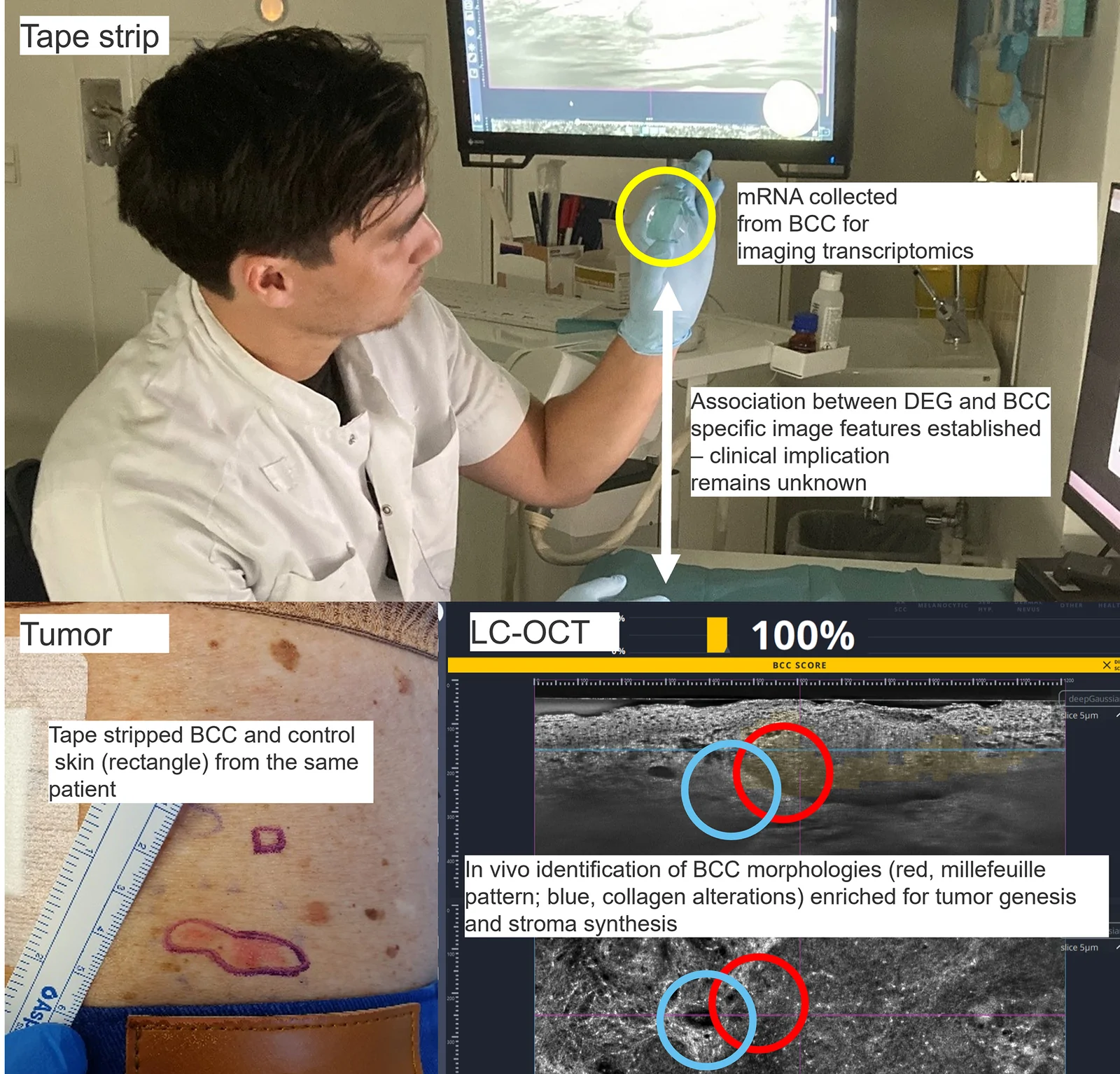
**Imaging transcriptomics key findings in BCC.** Top: tape-strip mRNA collected from the tumor. Bottom left: tape-stripped BCC and matched control skin. Bottom right: LC-OCT morphologies associated with DEGs, including the millefeuille pattern (red circle) (n = 5 DEGs) and collagen alterations (blue circle) (n = 9 DEGs) visualized in cross-section and en face. The overlapping region (shared area, n = 13 DEGs) indicates an interface between these features. Associated pathways were enriched for tumorigenesis (the millefeuille pattern in LC-OCT) and stromal synthesis/remodeling (collagen alterations in LC-OCT). Written informed consent for publication of the clinical image was obtained from the subject. BCC, basal cell carcinoma; DEG, differentially expressed genes; LC-OCT, line-field confocal optical coherence tomography.

The shared DEGs between the millefeuille pattern (tumor) and collagen alterations (peritumoral) warrant further investigation into their biological significance. Given that the image features are in close proximity to one another and often coexist in BCC (Figure 4), we speculate whether their associated genes could reflect the tumor-stroma environment or a more representative noninvasive DEG signature, but this remains to be elucidated.

### Strengths, future perspectives, and limitations

A key strength of this study’s approach is the integration of multiple non-invasive or minimally invasive technologies and the use of both expert assessment and high-throughput in-silico methods to identify gene expression levels associated with LC-OCT image features.

Tape stripping primarily samples the superficial epidermis, whereas BCC tumor nests are typically located in the lower epidermis and the dermis. The first strip probably mainly contains keratin and lipids. Later strips might yield keratinocytes from the upper granular layer and inflammatory cells. Thus, the biological significance of imaging-transcriptomic genes identified by tape stripping in relation to the underlying BCC remains to be established. This study was an initial step towards this goal. Although we focused on DEGs with an FDR threshold of 0.001 in this study, relaxing the FDR to 0.05 for the millefeuille pattern and collagen alterations revealed HH pathway-related genes, highlighting the potential of imaging transcriptomics in BCC (Supplementary Table S4). To further establish biological meaning, future imaging-transcriptomic studies could explore correlations between different optical BCC subtypes (superficial vs infiltrative) and gene expression levels obtained by tape stripping.

The pros and cons of in vivo and ex vivo approaches for gene expression analysis in BCC will likely be an area of interest for future studies. Tape stripping combined with in vivo imaging enables repeated, rapid tape sampling, is patient-tolerable, and links physical features to molecular pathways. Other techniques, such as ex vivo spatial transcriptomics, enable direct spatial correlation of gene expression with histologically defined tissue architecture using RNA-targeting probes (Yerly et al, 2022). The strengths of the latter include precise molecular profiling within a preserved histologic context, the ability to distinguish tumor and stromal compartments, and the assessment of deeper invasive tumor regions. However, the technology relies on tissue processing and histologic sectioning and is hence invasive compared with the non-invasive tape-stripping and imaging.

The study has some limitations. A major limitation is that mRNA collected from the skin surface was not validated against other methods, such as full-thickness biopsies or spatial methods such as immunohistochemistry, to draw more substantiated conclusions. Moreover, given the small sample size for the secondary outcome, the enrichment pathway findings should be interpreted with caution. We did not further investigate the DEGs associated with 5 out of 7 BCC image-specific features since they did not meet the FDR <0.001 threshold. Their failure to meet this FDR criteria is likely a result of their lower prevalence in the BCC dataset.

## Materials and Methods

### Study population and ethics

This study recruited human subjects referred to the Department of Dermatology at Bispebjerg Hospital. Patients over 18 years of age with a histology-verified BCC before or after in vivo imaging were prospectively enrolled (December 2024 and March 2025). The study was performed strictly in accordance with the Declaration of Helsinki, and written informed consent was obtained from all participants. The study received approval from the national ethics committee (H-24060543) and was registered on ClinicalTrials.gov (NCT06813833). In addition, the study was recorded in the University of Copenhagen’s research database, which contains personal data (ref. no. 514-1076/24-3000), and adhered to the European General Data Protection Regulation (2016/679). Written informed consent for publication of the clinical image was obtained from the subject.

### Data collection

Data collection included tape stripping and LC-OCT imaging of paired BCC and control skin from the same patient (Figure 1a). First, BCC and nearby control skin were encircled using a sterile surgical pen (Leonhard Lang, Innsbruck, Austria). Photographic documentation was performed with a scale bar and a digital camera to record lesion size and location. Tape stripping was then conducted on BCC and control skin using sterile hospital barrier tape (Mølnlycke, Gothenburg, Sweden), massaged for 20 seconds, and repeated twice at each site to ensure sufficient RNA yield. Each tape strip was immediately placed in a Petri dish, covered with a lid, and stored in an envelope to minimize light exposure before being transferred to a −80 °C freezer.

LC-OCT imaging of the same tape stripping sites was performed using a commercially available device (deepLive, DAMAE Medical, Paris, France). Briefly, the device transforms backscattered light from the skin layers into grey scale images using interferometry technology from OCT (Huang et al, 1991) combined with a confocal objective (numerical aperture 0.5) that provides images with an isotropic resolution of 1 μm at a depth of approximately 400–500 μm (Dubois et al, 2018a).

### Image feature analysis

A representative 3-dimensional image of each BCC and matched control skin was assessed for the presence of 44 established LC-OCT image features associated with the most common types of skin cancer, as previously described in detail (Jacobsen et al, 2024). All images were evaluated by a single investigator with more than 3.5 years of experience in LC-OCT and were assessed using the built-in proprietary computer software (DAMAE, deepLive) described elsewhere (Latriglia et al, 2023; Ogien et al, 2023).

### RNA extraction and sequencing

We cut the marked areas on thawed tape strips, combined same-site samples, and eluted RNA in 11 μL of RNase-free water using the miRNeasy Micro Kit (Qiagen) as described (Heerfordt et al, 2022). RNA quality was assessed with the Agilent 2100 Bioanalyzer and RNA 6000 Pico Assay Kit. Sequencing libraries were prepared with Takara Bio's SMART-Seq Total RNA Single Cell (ZapR Mammalian) protocol using 7 μL of RNA. Libraries were analyzed on the Agilent 2100 Bioanalyzer with the High Sensitivity DNA Kit and quantified with the KAPA Library Quantification Kit (Roche) on a 7500 Real-Time PCR System. RNA sequencing was performed on the NovaSeq 6000 (Illumina) with a 200-cycle run using the NovaSeq 6000 S4 Reagent Kit version 1.5 BCL files were converted to FASTQ format using bcl2fastq2 Conversion Software version 2.20 (Illumina). Read quality was assessed with FastQC (Babraham Bioinformatics). Next, we removed sequencing adapters, low-quality reads (Q <30), and reads shorter than 30 base pairs using AdapterRemoval version 2.3.2 (Lindgreen 2012). Alignment of reads was done to the human genome assembly GRCh38.p13 using the STAR aligner version 2.5.3a and the GENCODE Consortium release 33 assembly (Frankish et al, 2019). Only reads mapping to protein-coding genes were analyzed further.

### Primary outcome: Imaging transcriptomics DEGs

The study was designed as a hypothesis-generating imaging-transcriptomics investigation. The primary outcome was the identification of imaging transcriptomics DEGs, defined as genes that were shared between those associated with the presence of BCC and at least 1 BCC-specific image feature. Consequently, to identify BCC-specific image features, the presence versus absence for each predefined imaging feature was compared between paired BCC and control skin samples using McNemar’s test, which is designed for paired binary data (McNemar, 1947). Imaging features with a significance level of *P* < .001 were considered BCC-specific. Then, to identify shared DEGs, we used the edgeR package version 4.4.2 (Robinson et al, 2010), to perform 2 analyses: (i) comparing gene expression levels between paired BCC and control skin (Figure 1b, Step 1), and (ii) comparing gene expression levels between samples with and without individual BCC-specific image features (Figure 1b, Step 2 and Step 3). Genes identified in both analyses were considered shared DEGs (Figure 1b, Step 4).

A total of 19,522 genes were initially considered for differentially expressed gene (DEG) analysis. After trimmed mean of M-values (TMM) normalization and filtering of low-expression genes using filterByExpr(), gene counts were modeled using negative binomial generalized linear models fitted with the quasi-likelihood framework implemented in edgeR (glmQLFit), which is recommended for RNA-seq count data and provides robust error-rate control. The following models were fitted for each DEG analysis:

i) BCC: glmQLTest (gene counts ∼ pairID + group (BCC vs control)). The latter test was repeated for BCC versus control, leaving 1 pair out at each step. One DEG not identified in all BCC-control pair analyses (n = 53) was excluded from further analysis.
ii) glmQLTest (gene counts ∼ pairID + single BCC-specific image feature (presence vs absence))

Pair ID (ie, paired BCC and control skin samples from the same patient) was included as a blocking factor in the DEG analysis. This decision was based on principal component analysis, which identified pair ID as a potential confounder (Supplementary Figure S2). The principal component analysis showed that samples from the same patient clustered more closely together than samples from different patients, indicating a strong patient-specific confounding effect on gene expression. principal component analysis was performed using the prcomp() function, and potential confounding variables evaluated included RNA sampling batch, extraction date, lesion size, age, sex, pair ID, patient ID, lesion ID, anatomical site, and study group.

DEGs with false discovery rate (FDR) values adjusted by the Benjamini–Hochberg method in edgeR (Benjamini and Hochberg, 1995) are presented in the manuscript. Given the exploratory nature of the study and to minimize type I errors, a stringent FDR threshold of <0.001 was applied to identify imaging-transcriptomic DEGs, which were included in the pathway enrichment analyses and literature validation. For transparency, all genes meeting an FDR threshold of <0.05 for each BCC-specific image feature were deposited in a public repository. Gene expression changes are reported as log_2_ fold change (log_2_FC), where a log_2_FC of 1 represents a 2-fold change in expression.

### Secondary outcome: Pathways enrichment analysis and literature validation

The shared DEGs between BCC and BCC-specific image features were further assessed using complementary analysis with the Kyoto Encyclopedia of Genes and Genomes database and the Gene Ontology (GO) cellular, molecular, and biological categories using ShinyGo (Ge et al, 2020), an online available database (https://bioinformatics.sdstate.edu/go/). The top pathways, based on effect size from the fold enrichment output provided by the ShinyGo software, were reported. Next, to determine whether the shared DEGs had been previously reported in BCC or other skin conditions, a literature validation was performed using PubMed and Google Scholar.

### Power calculation

Given the exploratory nature of the study, the power calculation used to determine the required sample size for patient recruitment was based on only 1 of the analyses of the study, namely the comparison of gene expression levels between BCC and paired control skin (Figure 1b, Step 1). That analysis was based on pilot data and the ssizeRNA method (Bi and Liu, 2016), indicated that a significant difference in gene expression (fold change = 2, power = 0.80, FDR <0.05) could be detected between 2 groups of 50 paired samples.

### Statistics

All statistical tasks were performed using R version 4.4.1 (R Core Team, 2021) and International Business Machines (IBM) Statistical Package for the Social Sciences (SPSS) Statistics version 29.0 (IBM Corporation, 2023).

## Conclusion

This exploratory imaging transcriptomic study suggests the feasibility of tape stripping for non-invasive transcriptomic profiling of BCC and the utility of coupling LC-OCT morphology with gene expression data. We identified 27 DEGs shared between BCC, of which 5 were uniquely associated with the millefeuille pattern and 9 were specific to collagen alterations. These findings offer insight into the potential benefit of integrating imaging and molecular approaches to deepen our understanding of BCC pathobiology in vivo.

## Ethics Statement

The study was performed strictly in accordance with the Declaration of Helsinki, and written, informed consent was obtained from all participants. The study received approval from the national ethics committee (H-24060543) and was registered on ClinicalTrials.gov (NCT06813833). In addition, the study is recorded in the University of Copenhagen’s research database, which contains personal data (ref. no. 514-1076/24-3000), and adheres to the European General Data Protection Regulation (2016/679). The study received approval from the national ethics committee (H-24060543) and was registered on ClinicalTrials.gov (NCT06813833).

## Data availability statement

Datasets related to this article can be found at https://data.mendeley.com/preview/3tgr98pp4x?a=c9b51171-f9dd-4af6-883b-541143b7ef2d, hosted at Mendeley Data. (Jacobsen, 2026)

## ORCIDs

Kevin Jacobsen: http://orcid.org/0000-0002-8577-6638

Olivia Luxford Meyer: http://orcid.org/0000-0002-7842-5581

Stine Bøttcher Jacobsen: http://orcid.org/0000-0002-6884-2701

Vinzent Kevin Ortner: http://orcid.org/0000-0001-9708-6717

Jeppe Dyrberg Andersen: http://orcid.org/0000-0002-4395-5232

Martin Glud: http://orcid.org/0000-0003-2173-0769

Merete Haedersdal: http://orcid.org/0000-0003-1250-2035

Katrine Elisabeth Karmisholt: http://orcid.org/0000-0001-8511-3634

Cyril Maliakal: http://orcid.org/0009-0000-6488-2055

Niels Morling: http://orcid.org/0000-0002-9463-5087

Peter Alshede Philipsen: http://orcid.org/0000-0002-9656-733X

Aditi Sahu: http://orcid.org/0000-0003-0004-3839

## Conflict of Interest

The authors state no conflict of interest.

## References

[bib1] Abdalla B.M.Z., Reiter O., Godwin K., Gallagher R., Monnier J., dos Reis Zuniga R.D. (2025). Noninvasive multimodal imaging and its role in diagnosing skin lesions in dermatology: A systematic review and meta-analysis. Am J Clin Dermatol.

[bib2] Adase C., Walker M., Shi J., McGhee J., Holscher T., Kim M. (2021). 058 Identification of novel gene classifiers to non-invasively diagnose non-melanoma skin cancer. J Invest Dermatol.

[bib3] Aerts H.J.W.L., Velazquez E.R., Leijenaar R.T.H., Parmar C., Grossmann P., Carvalho S. (2014). Decoding tumour phenotype by noninvasive imaging using a quantitative radiomics approach. Nat Commun.

[bib4] Alkemade H.A., Molhuizen H.O., van Vlijmen-Willems I.M., van Haelst U.J., Schalkwijk J. (1993). Differential expression of SKALP/elafin in human epidermal tumors. Am J Pathol.

[bib5] Beck L.A., Cork M.J., Amagai M., De Benedetto A., Kabashima K., Hamilton J.D. (2022). Type 2 inflammation contributes to skin barrier dysfunction in atopic dermatitis. JID Innov.

[bib72] Benjamini Y., Hochberg Y. (1995). Controlling the false discovery rate: a practical and powerful approach to multiple testing. Journal of the Royal Statistical Society.

[bib6] Bi R., Liu P. (2016). Sample size calculation while controlling false discovery rate for differential expression analysis with RNA-sequencing experiments. BMC Bioinform.

[bib7] Bigelow R.L.H., Chari N.S., Undén A.B., Spurgers K.B., Lee S., Roop D.R. (2004). Transcriptional regulation of *bcl-2* mediated by the sonic hedgehog signaling pathway through gli-1. J Biol Chem.

[bib8] Cappilli S., Dejonckheere G., Hajjar N., Cinotti E., Tognetti L., Perez-Anker J. (2022). Line-field confocal optical coherence tomography: a case on the importance of full-lesion examination for basal cell carcinoma. Int J Dermatol.

[bib9] Chelvanambi M., Weinstein A.M., Storkus W.J. (2020). IL-36 signaling in the tumor microenvironment. Adv Exp Med Biol.

[bib10] Cinotti E., Bertello M., Dragotto M., Cartocci A., Tognetti L., Cappilli S. (2023). Comparison of reflectance confocal microscopy and line-field optical coherence tomography for the identification of basal cell carcinoma. J Eur Acad Dermatol Venereol.

[bib11] Dachani S.R., Kaleem M., Mujtaba M.D.A., Mahajan N., Ali S.A., Almutairy A.F. (2024). A comprehensive review of various therapeutic strategies for the management of skin cancer. ACS Omega.

[bib12] Dai J., Lin K., Huang Y., Lu Y., Chen W.-Q., Zhang X.-R. (2018). Identification of critically carcinogenesis-related genes in basal cell carcinoma. Onco Targets Ther.

[bib13] Dubois A., Levecq O., Azimani H., Davis A., Ogien J., Siret D. (2018). Line-field confocal time-domain optical coherence tomography with dynamic focusing. Opt Express.

[bib14] Dubois A., Levecq O., Azimani H., Siret D., Barut A., Suppa M. (2018). Line-field confocal optical coherence tomography for high-resolution noninvasive imaging of skin tumors. J Biomed Opt.

[bib15] Eckert R.L., Broome A.-M., Ruse M., Robinson N., Ryan D., Lee K. (2004). S100 proteins in the epidermis. J Invest Dermatol.

[bib16] Eichberger T., Sander V., Schnidar H., Regl G., Kasper M., Schmid C. (2006). Overlapping and distinct transcriptional regulator properties of the GLI1 and GLI2 oncogenes. Genomics.

[bib17] Ferrante di Ruffano L., Dinnes J., Deeks J.J., Chuchu N., Bayliss S.E., Davenport C. (2018). Optical coherence tomography for diagnosing skin cancer in adults. Cochrane Database Syst Rev.

[bib18] Frankish A., Diekhans M., Ferreira A.-M., Johnson R., Jungreis I., Loveland J. (2019). GENCODE reference annotation for the human and mouse genomes. Nucleic Acids Res.

[bib19] Freeman B.D., Buchman T.G. (2001). Interleukin-1 receptor antagonist as therapy for inflammatory disorders. Expert Opin Biol Ther.

[bib20] Fullen D.R., Garrisi A.J., Sanders D., Thomas D. (2008). Expression of S100A6 protein in a broad spectrum of cutaneous tumors using tissue microarrays. J Cutan Pathol.

[bib21] Ge S.X., Jung D., Yao R. (2020). ShinyGO: a graphical gene-set enrichment tool for animals and plants. Bioinformatics.

[bib73] Haq R, Yokoyama S, Hawryluk EB, Jönsson GB, Frederick DT, McHenry K (2013). BCL2A1 is a lineage-specific antiapoptotic melanoma oncogene that confers resistance to BRAF inhibition. Proc Natl Acad Sci.

[bib22] Heerfordt I.M., Andersen J.D., Philipsen P.A., Langhans L., Tvedebrink T., Schmidt G. (2022). Detection of cutaneous malignant melanoma using RNA sampled by tape strips: A study protocol. PLOS One.

[bib23] Hu T., Todberg T., Andersen D., Danneskiold-Samsøe N.B., Hansen S.B.N., Kristiansen K. (2022). Profiling the atopic dermatitis epidermal transcriptome by tape stripping and BRB-seq. Int J Mol Sci.

[bib24] Huang D., Swanson E.A., Lin C.P., Schuman J.S., Stinson W.G., Chang W. (1991). Optical coherence tomography. Science.

[bib74] IBM Corporation (2023). IBM SPSS Statistics for Windows, Version 29.0.

[bib25] Jackson B., Tilli C.M.L.J., Hardman M.J., Avilion A.A., MacLeod M.C., Ashcroft G.S. (2005). Late cornified envelope family in differentiating epithelia--response to calcium and ultraviolet irradiation. J Invest Dermatol.

[bib75] Jacobsen K. (2026). Imaging transcriptomics. Mendeley Data.

[bib26] Jacobsen K., Ortner V.K., Wenande E., Sahu A., Paasch U., Haedersdal M. (2024). Line-field confocal optical coherence tomography in dermato-oncology: A literature review towards harmonized histopathology-integrated terminology. Exp Dermatol.

[bib27] Jasmine F., Argos M., Khamkevych Y., Islam T., Rakibuz-Zaman M., Shahriar M. (2024). Molecular profiling and the interaction of somatic mutations with transcriptomic profiles in non-melanoma skin cancer (NMSC) in a population exposed to arsenic. Cells.

[bib28] Kaporis H.G., Guttman-Yassky E., Lowes M.A., Haider A.S., Fuentes-Duculan J., Darabi K. (2007). Human basal cell carcinoma Is Associated with Foxp3+ T cells in a Th2 Dominant microenvironment. J Invest Dermatol.

[bib29] Kumar M., Srivastava G., Kaur J., Assi J., Alyass A., Leong I. (2015). Prognostic significance of cytoplasmic S100A2 overexpression in oral cancer patients. J Transl Med.

[bib30] Latriglia F., Ogien J., Tavernier C., Fischman S., Suppa M., Perrot J.-L. (2023). Line-field confocal optical coherence tomography (LC-OCT) for skin imaging in dermatology. Life (Basel).

[bib31] Li G., Gu L., Zhao F., Hu Y., Wang X., Zeng F. (2023). WFDC12-overexpressing contributes to the development of atopic dermatitis via accelerating ALOX12/15 metabolism and PAF accumulation. Cell Death Dis.

[bib32] Li S., Zhou B. (2022). A review of radiomics and genomics applications in cancers: the way towards precision medicine. Radiat Oncol.

[bib33] Lindgreen S. (2012). AdapterRemoval: easy cleaning of next-generation sequencing reads. BMC Res Notes.

[bib34] Liu Z., Duan T., Zhang Y., Weng S., Xu H., Ren Y. (2023). Radiogenomics: a key component of precision cancer medicine. Br J Cancer.

[bib35] MacDonald M., Bergeron S., Darwich R., Daigle P., Burnier J., Burnier M.N. (2019). Is elafin part of a protective system in basal cell carcinomas of the eyelid?. Invest Ophthalmol Vis Sci.

[bib36] Majoros H., Ujfaludi Z., Borsos B.N., Hudacsek V.V., Nagy Z., Coin F. (2019). SerpinB2 is involved in cellular response upon UV irradiation. Sci Rep.

[bib37] Maroufy V., Shah P., Asghari A., Deng N., Le R.N.U., Ramirez J.C. (2020). Gene expression dynamic analysis reveals co-activation of Sonic Hedgehog and epidermal growth factor followed by dynamic silencing. Oncotarget.

[bib38] Matas-Nadal C., Bech-Serra J.J., Gatius S., Gomez X., Ribes-Santolaria M., Guasch-Vallés M. (2023). Biomarkers found in the tumor interstitial fluid may help explain the differential behavior among keratinocyte carcinomas. Mol Cell Proteomics.

[bib39] Mattiola I., Tomay F., De Pizzol M., Silva-Gomes R., Savino B., Gulic T. (2019). The macrophage tetraspan MS4A4A enhances dectin-1-dependent NK cell–mediated resistance to metastasis. Nat Immunol.

[bib40] McNemar Q. (1947). Note on the sampling error of the difference between correlated proportions or percentages. Psychometrika.

[bib41] Meng L., He X., Hong Q., Qiao B., Zhang X., Wu B. (2021). CCR4, CCR8, and P2RY14 as prognostic factors in head and neck squamous cell carcinoma are involved in the remodeling of the tumor microenvironment. Front Oncol.

[bib42] Minaei E., Mueller S.A., Ashford B., Thind A.S., Mitchell J., Perry J.R. (2022). Cancer progression gene expression profiling identifies the urokinase plasminogen activator receptor as a biomarker of metastasis in cutaneous squamous cell carcinoma. Front Oncol.

[bib43] Moufarrij S., Filippova O., Da Cruz Paula A.D.C., Heredia J.B., Green H., Broach V. (2025). Molecular and microenvironmental landscapes of human papillomavirus-independent invasive squamous cell carcinoma of the vulva. Int J Gynecol Cancer.

[bib44] Mtimet L., Boussingault L., Aktas D., Fontaine M., Orte Cano C., Diet G. (2025). Line-field confocal optical coherence tomography of basal cell carcinoma: a retrospective study on diagnostic performance. J Eur Acad Dermatol Venereol.

[bib45] Ogien J., Tavernier C., Fischman S., Dubois A. (2023). Line-field confocal optical coherence tomography (LC-OCT): principles and practical use. Ital J Dermatol Venerol.

[bib46] Pan H., Wang X., Huang W., Dai Y., Yang M., Liang H. (2020). Interferon-induced Protein 44 correlated with immune infiltration serves as a potential prognostic indicator in head and neck squamous cell carcinoma. Front Oncol.

[bib47] Pant A., Jain A., Chen Y., Patel K., Saleh L., Tzeng S. (2024). The CCR6–CCL20 axis promotes regulatory T-cell glycolysis and immunosuppression in tumors. Cancer Immunol Res.

[bib48] Pereira M.S.F., Carr D.R., Gatti-Mays M.E., Olsen M.R., Setty B.A., Shahwan K.T. (2022). Natural killer cell recognition and control of epithelial cancers. Cancer J.

[bib49] Peris K., Fargnoli M.C., Garbe C., Kaufmann R., Bastholt L., Seguin N.B. (2019). Diagnosis and treatment of basal cell carcinoma: European consensus–based interdisciplinary guidelines. Eur J Cancer.

[bib50] Ramchatesingh B., Martinez Villarreal A., Lefrançois P., Gantchev J., Sivachandran S., Abou Setah S. (2025). Targeting PRAME directly or via EZH2 inhibition overcomes retinoid resistance and represents a novel therapy for keratinocyte carcinoma. Mol Oncol.

[bib51] Ren F., Su F., Ning H., Wang Y., Geng Y., Feng Y. (2013). SIPAR negatively regulates STAT3 signaling and inhibits progression of melanoma. Cell Signal.

[bib52] Rittié L., Kansra S., Stoll S.W., Li Y., Gudjonsson J.E., Shao Y. (2007). Differential ErbB1 Signaling in squamous Cell versus basal cell carcinoma of the Skin. Am J Pathol.

[bib53] Robinson M.D., McCarthy D.J., Smyth G.K. (2010). edgeR: a Bioconductor package for differential expression analysis of digital gene expression data. Bioinformatics.

[bib54] Rogers H.W., Weinstock M.A., Feldman S.R., Coldiron B.M. (2015). Incidence estimate of nonmelanoma skin cancer (keratinocyte carcinomas) in the U.S, population, 2012. JAMA Dermatol.

[bib55] Rubin A.I., Chen E.H., Ratner D. (2005). Basal-cell carcinoma. N Engl J Med.

[bib56] Ruini C., Schuh S., Gust C., Kendziora B., Frommherz L., French L.E. (2021). Line-field optical coherence tomography: in vivo diagnosis of basal cell carcinoma subtypes compared with histopathology. Clin Exp Dermatol.

[bib57] Rupa D., Chuang H.W., Hu C.E., Su W.M., Wu S.R., Lee H.S. (2024). ACSL4 upregulates IFI44 and IFI44L expression and promotes the proliferation and invasiveness of head and neck squamous cell carcinoma cells. Cancer Sci.

[bib58] Sahu A., Kose K., Kraehenbuehl L., Byers C., Holland A., Tembo T. (2022). In vivo tumor immune microenvironment phenotypes correlate with inflammation and vasculature to predict immunotherapy response. Nat Commun.

[bib59] Schmults C.D., Blitzblau R., Aasi S.Z., Alam M., Amini A., Bibee K. (2023). Basal cell skin cancer, version 2.2024, NCCN clinical practice guidelines in oncology. J Natl Compr Canc Netw.

[bib60] Shin J.-M., Chang I.-K., Lee Y.-H., Yeo M.-K., Kim J.-M., Sohn K.-C. (2016). Potential role of S100A8 in cutaneous squamous cell carcinoma differentiation. Ann Dermatol.

[bib61] Sølberg J.B.K., Quaade A.S., Jacobsen S.B., Andersen J.D., Kampmann M.-L., Morling N. (2022). The transcriptome of hand eczema assessed by tape stripping. Contact Dermat.

[bib62] Suppa M., Fontaine M., Dejonckheere G., Cinotti E., Yélamos O., Diet G. (2021). Line-field confocal optical coherence tomography of basal cell carcinoma: a descriptive study. J Eur Acad Dermatol Venereol.

[bib63] Venables Z.C., Wakkee M. (2024). Basal cell carcinoma incidence trends: can we stop the surge?. Br J Dermatol.

[bib64] Yang F., Ma J., Zhu D., Wang Z., Li Y., He X. (2023). The role of S100A6 in human diseases: molecular mechanisms and therapeutic potential. Biomolecules.

[bib65] Yerly L., Pich-Bavastro C., Di Domizio J., Wyss T., Tissot-Renaud S., Cangkrama M. (2022). Integrated multi-omics reveals cellular and molecular interactions governing the invasive niche of basal cell carcinoma. Nat Commun.

[bib71] Yoshimura T., Manabe C., Nagumo J-I., Nagahaa T., Sato T., Murakami S. (2023). Taurine accelerates the synthesis of ceramides and hyaluronic acid in cultured epidermis and dermal fibroblasts. Exp Ther Med.

[bib66] Yoshioka M., Sawada Y., Saito-Sasaki N., Yoshioka H., Hama K., Omoto D. (2021). High S100A2 expression in keratinocytes in patients with drug eruption. Sci Rep.

[bib67] Zhang M., Chen T., Lu X., Lan X., Chen Z., Lu S. (2024). G protein-coupled receptors (GPCRs): advances in structures, mechanisms and drug discovery. Signal Transduct Target Ther.

[bib68] Zhang M., Wang X., Guo F., Jia Q., Liu N., Chen Y. (2019). Cdc42 deficiency leads to epidermal barrier dysfunction by regulating intercellular junctions and keratinization of epidermal cells during mouse skin development. Theranostics.

[bib69] Zilberg C., Lyons J.G., Gupta R., Damian D.L. (2023). The immune microenvironment in basal cell carcinoma. Ann Dermatol.

[bib70] Zou D.-D., Sun Y.-Z., Li X.-J., Wu W.-J., Xu D., He Y.-T. (2023). Single-cell sequencing highlights heterogeneity and malignant progression in actinic keratosis and cutaneous squamous cell carcinoma. eLife.

