# Supplementary material for: Imaging transcriptomics in basal cell carcinoma: Coupling in vivo tumor morphology with gene expression
Source: JID Innov. 2026 Jul 28;6(5):100519. doi: 10.1016/j.xjidi.2026.100519 (PMC13571268; doi:10.1016/j.xjidi.2026.100519)
Supplement: Supplementary file 1 [file mmc1.pdf]

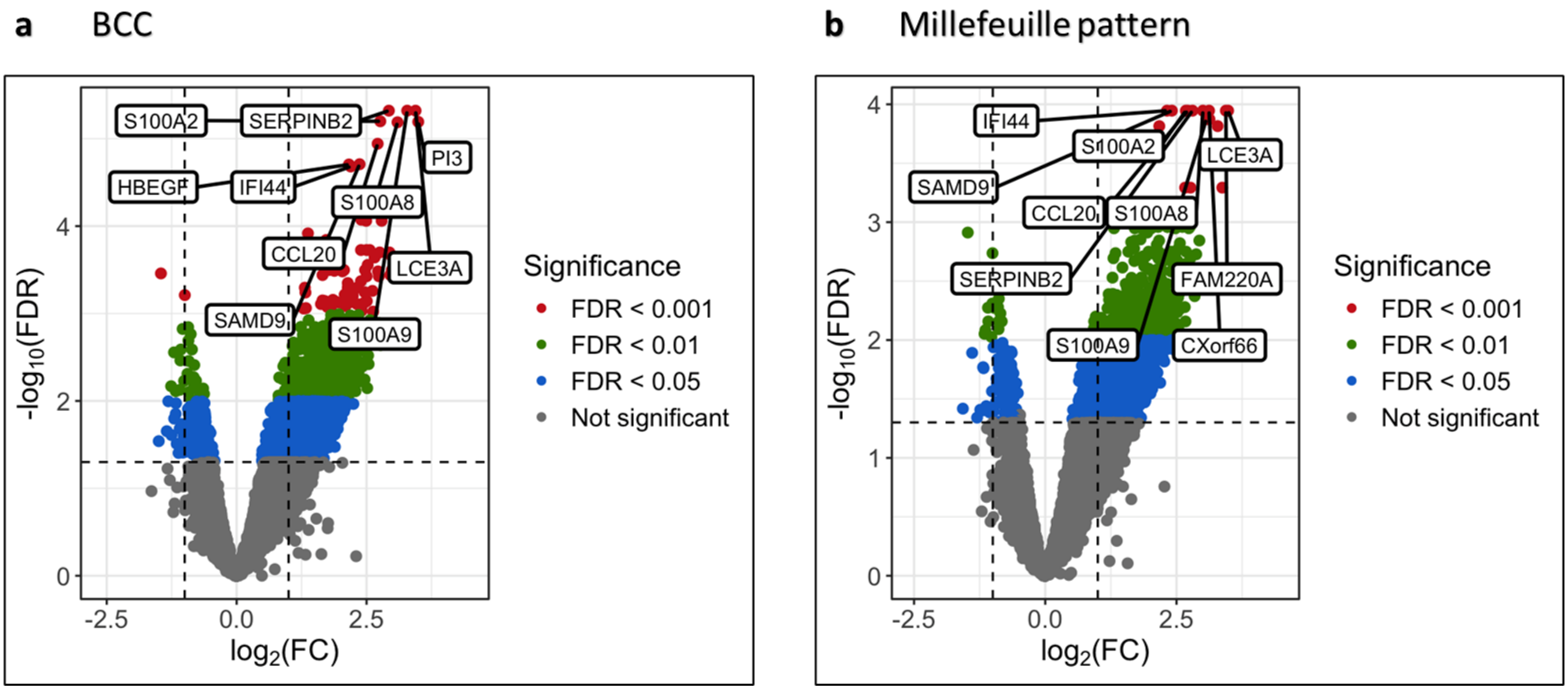

Supplementary Figure 1: Differentially expressed genes in basal cell carcinoma and the millefeuille pattern. Differences in gene expression between basal cell carcinoma (BCC) and healthy skin (A) and between the presence of the millefeuille pattern and absence (B). Similar analyses were performed for all BCC specific image features. On the x-axis, $\log_2$ (fold-change (FC)) indicates a two-fold change of gene expression. On the y-axis, -$\log_{10}$(false discovery rate (FDR)). Red dots represent FDR < 0.001, green dots FDR < 0.01, blue dots FDR < 0.05, and grey dots FDR ≥ 0.05, i.e., not significant.

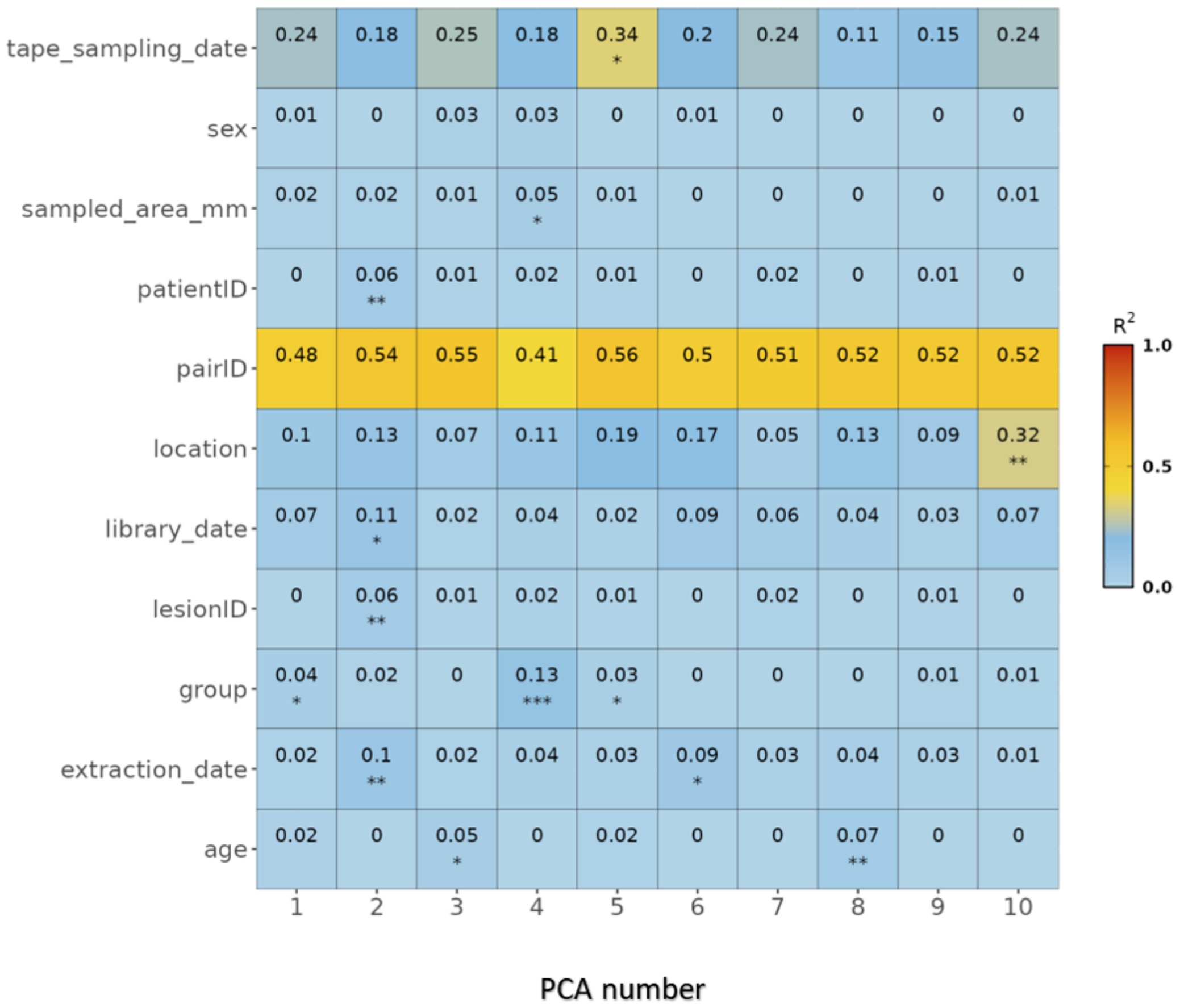

Supplementary Figure 2: Variance in principal components explained by study variables

Associations were evaluated between the first 10 principal components (PC) and study metadata: patient identification (patientID), lesion identification (lesionID), diagnosis group (basal cell carcinoma and healthy skin), mRNA extraction date, library preparation date, patient age, sex, tape sampling date, sampled skin area in mm², anatomical location, and the identification number of each paired basal cell carcinoma and healthy skin (pairID). Coefficients (R^2) indicate the proportion of variance in each principal component explained by the variables, while * denotes which associations are statistically significant. PairID showed consistently high R^2 across all 10 principal components, indicating that paired samples account for a large proportion of the variance in the PC analysis and are more similar to each other than to samples from other pairs. It was included as a blocking factor in the differential expression design matrix to prevent false-positive results. In contrast, low R^2 was observed for intra-patient (patientID) and inter-group (lesionID) comparisons across the principal components, supporting the assumption that each basal cell carcinoma can be treated as an independent sample, even in patients with multiple basal cell carcinomas.

Supplementary Table 1. Patient and basal cell carcinoma characteristics

**Patients**	**(n=34)**
Female, n (%)	22 (64.7)
Age (y), mean ± SD	61.0 ± 12.9
Number of BCCs, n (%)	
1	20 (58.8)
2	9 (26.5)
3	5 (14.7)

**Basal cell carcinomas**	**(n=53)**
Location, n (%)	
Head and Neck	28 (53)
Trunk	3 (6)
Back	12 (23)
Upper extremities	2 (4)
Lower extremities	8 (15)
Clinical tumor size, n (%)	
< 2 cm	49 (92)
≥2cm	4 (8)
Subtypes, n (%)	
sBCC	6 (11.3)
nBCC	45 (84.9)
iBCC	2 (4)

Abbreviations: sBCC, superficial basal cell carcinoma; nBCC, nodular basal cell carcinoma; iBCC, infiltrative basal cell carcinoma; y, years. Sum of percentages may not total 100% due to rounding.

Supplementary Table 2: Differentially expressed genes associated with basal cell carcinoma

Gene name	Log2<br>Fold Change	False discovery rate*
PI3	11.2× (3.49)	6.4E-06
LCE3A	10.8× (3.44)	4.8E-06
S100A9	9.8× (3.28)	4.8E-06
DEFB131B	8.8× (3.13)	3.2E-04
S100A8	8.6× (3.10)	6.5E-06
FAM220A	7.9× (2.98)	2.2E-04
CXCL10	7.7× (2.96)	3.6E-04
RNF103-CHMP3	7.7× (2.94)	2.0E-04
SERPINB2	7.6× (2.93)	4.8E-06
SPRR2A	6.9× (2.79)	8.7E-05
S100A2	6.8× (2.76)	6.3E-06
S100A12	6.8× (2.76)	4.7E-05
GPR183	6.7× (2.75)	2.0E-04
CLEC4A	6.7× (2.74)	3.7E-04
FAM72B	6.6× (2.72)	3.3E-04
CCL20	6.6× (2.71)	1.1E-05
SPRR2G	6.4× (2.68)	5.9E-05
DEFB103A	6.3× (2.65)	2.3E-04
TAS2R19	6.2× (2.63)	9.5E-04
ARL2BP	6.1× (2.61)	5.5E-04
MMP1	5.9× (2.56)	1.9E-04
LCE3D	5.8× (2.52)	2.8E-04
MMP3	5.7× (2.51)	7.2E-04
CLEC2A	5.7× (2.50)	3.7E-04
MMP10	5.7× (2.50)	1.9E-04
IL36G	5.6× (2.49)	8.7E-05
MS4A4A	5.5× (2.47)	3.2E-04
FCER1G	5.5× (2.46)	5.7E-04
P2RY14	5.5× (2.46)	7.5E-04
SPRR2B	5.5× (2.45)	8.5E-05
CR589904.2	5.3× (2.41)	7.8E-04
GUCA1C	5.3× (2.41)	9.3E-04
BCL2A1	5.3× (2.40)	8.5E-05

S100A6	5.3× (2.39)	1.9E-04
GCG	5.3× (2.39)	4.3E-04
SAMD9	5.2× (2.37)	2.0E-05
HSPA1A	5.0× (2.32)	5.1E-04
C1orf52	5.0× (2.31)	7.8E-04
LCE3E	4.7× (2.24)	6.2E-04
IFI44	4.6× (2.20)	2.1E-05
CD14	4.5× (2.16)	7.5E-04
HBEGF	4.5× (2.16)	2.0E-05
TMPRSS11D	4.4× (2.14)	4.5E-04
SLC38A2	4.4× (2.14)	5.1E-04
DHRS9	4.3× (2.11)	6.0E-04
ADAM20	4.3× (2.11)	7.8E-04
SRFBP1	4.3× (2.10)	8.2E-04
KRT6C	4.2× (2.08)	6.1E-04
FMO2	4.2× (2.07)	3.2E-04
WFDC12	4.1× (2.04)	3.3E-04
HAL	4.0× (1.99)	7.5E-04
ME1	3.7× (1.89)	7.2E-04
ADAM32	3.7× (1.88)	3.2E-04
RPLP2	3.7× (1.87)	7.5E-04
CHCHD7	3.6× (1.85)	7.0E-04
CTSL	3.5× (1.81)	8.7E-04
IL1RN	3.4× (1.78)	3.2E-04
DSC2	3.4× (1.76)	2.3E-04
SERPINB1	3.4× (1.75)	8.4E-04
GLS	3.3× (1.72)	1.4E-04
HEPHL1	3.2× (1.70)	7.2E-04
MAP3K8	3.1× (1.65)	2.2E-04
WSB1	3.1× (1.65)	3.6E-04
SELENOS	3.1× (1.65)	7.1E-04
ZNF165	3.1× (1.62)	7.8E-04
FNBP4	2.6× (1.37)	1.2E-04
GSPT1	2.5× (1.33)	8.7E-04
OXSR1	2.5× (1.32)	5.8E-04
NFKBIZ	2.5× (1.31)	5.1E-04
NPR2	2.4× (1.29)	8.8E-04

BAHCC1	0.5× (-1.00)	6.2E-04
PERCC1	0.4× (-1.46)	3.5E-04

Differentially expressed genes (DEGs) are sorted by the change in gene expression. Only DEGs with a false discovery rate of 0.001 after Benjamini–Hochberg corrections are presented.

Supplementary Table 3: Image features and comparison of frequencies between basal cell carcinoma and control skin

Skin layer	Image feature	BCC (n=53), n (%)	Control (n=53), n (%)	P-value
Stratum corneum	Hyperkeratosis	7 (13.2)	3 (5.7)	0.289
	Scales	6 (11.3)	4 (7.5)	0.727
	Parakeratosis	4 (7.5)	0 (0)	0.125
	Ulceration	7 (13.2)	0 (0)	0.016
	Crust	1 (1.9)	0 (0)	1.0
The viable epidermis	Epidermal acanthosis	5 (9.4)	0(0)	0.063
	**Epidermal atrophy**	**29 (54.7)**	**0 (0)**	**<0.001**
	Achantholysis	3 (5.7)	0 (0)	0.250
	Tumor budding	1 (1. 9)	0 (0)	1.0
	Adnexal involvement	2 (3.8)	4 (7.5)	0.687
	Epidermal dysplasia	3 (5.7)	2 (3.8)	1.0
	Severe dysplasia	0 (0.0)	2 (3.8)	0.5
	Moderate dysplasia	3 (5.7)	1 (1.9)	0.625
	Mild dysplasia	3 (5.7)	1 (1.9)	0.625
	Keratin plug	5 (9.4)	5 (9.4)	1.0
	**Lobule connected to epidermis**	**19 (35.8)**	**0 (0)**	**<0.001**
	String of pearls	7 (13.2)	0 (0)	0.016
	Non-edged papillae	2 (3.8)	0 (0)	0.500
	Edged papillae	15 (28.3)	22 (41.5)	0.248
	Typical honeycomb pattern	44 (83.0)	48 (90.6)	0.424
	Medusa-like structure	0 (0.0)	0 (0)	**
	Presence of atypical keratinocyte	11 (20.8)	9 (17.0)	0.791
	Suprabasal hyperpigmented keratinocyte	3 (5.7)	8 (15.1)	0.180
	Epidermal bright structures/cells	52 (98.1)	53 (100)	1.0
	Presence of dendritic cells	1 (1.9)	1 (1.9)	1.0
	Presence of pagetoid cells	0 (0)	1 (1.9)	1.0
	Presence of nest of atypical melanocytes [General]	0 (0)	1 (1.9)	1.0
	Epidermal nest of atypical melanocytes	0 (0)	0 (0)	**
	Presence of single atypical melanocytes	0 (0)	0 (0)	**
DEJ	Well-defined dermo-epidermal junction	49 (92.5)	52 (98.1)	0.375
	Irregular dermo-epidermal junction	4 (7.5)	1 (1.9)	0.375
	**Collagen alterations**	**42 (79.2)**	**0 (0)**	**<0.001**

	Dilated vessels	31 (58.5)	48 (90.6)	<0.001
	Glomerular vessels	26 (49.1)	20 (37.7)	0.377
	Dermal bright particles	3 (66.0)5	53 (100)	<0.001
	Broad strands	0 (0)	0 (0)	**
	Lobules	-	-	-
	**Lobule not connected to epidermis**	**35 (66.0)**	**0 (0)**	**<0.001**
	Shoal of fish	5(9.4)	0 (0)	0.063
	**Clefting**	**43 (81.1)**	**0 (0)**	**<0.001**
	**Millefeuille pattern**	**48 (90.6)**	**0 (0)**	**<0.001**
	Lobule [palisading]	2 (3.8)	0 (0)	0.5
Dermis	**Intratumoral hyporeflectivity**	**9 (17.0)**	**0 (0)**	**0.004**
	Keratin cyst/pearl	3 (5.7)	0 (0)	0.250
	Dermal nest of atypical melanocytes	0 (0)	0 (0)	**

McNemar's test was performed for each line-field confocal optical coherence image feature to compare frequencies in basal cell carcinoma and paired control skin. ** statistical test not applicable due to lack of variation between groups. DEJ, dermal epidermal junction. Features significantly associated with BCC are shown

Supplementary Table 4: Differentially expressed genes (DEGs) associated with the millefeuille pattern and collagen alterations that are present in the KEGG Hedgehog (HH) pathway (FDR < 0.05).

DEGs	Present on Hedgehog pathway map*	Interpreted Function in Pathway
**Millefeuille pattern**		
HHIP	Yes	HH downstream target
CSNK1A1	Yes (CK1)	SMO regulation
CSNK1G1	Yes (CK1)	SMO regulation
CSNK1G2	Yes (CK1)	SMO regulation
BCL2A1	Yes (BCL2)	HH downstream target
**Collagen alterations**		
CSNK1A1	Yes (CK1)	SMO regulation
CSNK1G1	Yes (CK1)	SMO regulation
BCL2A1	Yes (BCL2)	HH downstream target

*https://www.kegg.jp/entry/hsa04340.
